# Women who smoke during pregnancy are more likely to be referred to an obstetrician during pregnancy and birth: results from a cohort study

**DOI:** 10.1186/s12884-022-04808-7

**Published:** 2022-06-13

**Authors:** S. Weiland, L.L. Peters, M.Y. Berger, J.J.H.M. Erwich, D.E.M.C. Jansen

**Affiliations:** 1 Department of General Practice and Elderly Care Medicine, University of Groningen, University Medical Center Groningen, Hanzeplein 1, 9713 GZ Groningen, The Netherlands; 2 Department of Midwifery Science AVAG, Amsterdam UMC, Vrije Universiteit Amsterdam, Amsterdam Public Health Research Institute, Amsterdam, The Netherlands; 3 Department of Obstetrics and Gynecology, University of Groningen, University Medical Center Groningen, Groningen, The Netherlands

**Keywords:** Prenatal care, Maternal healthcare utilization, Smoking, Referral and consultation

## Abstract

**Background:**

Women who smoke during pregnancy make less use of prenatal care; the relation of smoking behavior with the use of other forms of maternal healthcare is unknown. The objective of this study is to investigate the association between women’s smoking behavior and their use of healthcare during pregnancy, birth and six weeks postpartum.

**Methods:**

We analyzed data from the Dutch Midwifery Case Registration System (VeCaS), period 2012–2019. We included women with a known smoking status, singleton pregnancies, and who had their first appointment before 24 weeks of gestation with the primary care midwife. We compared three groups: non-smokers, early stoppers (stopped smoking in the first trimester), and late- or non-stoppers (stopped smoking after the first trimester or continued smoking). Descriptive statistics were used to report maternal healthcare utilization (during pregnancy, birth and six weeks postpartum), statistical differences between the groups were calculated with Kruskal–Wallis tests. Multivariable logistic regression was conducted to assess the association between smoking behavior and referrals to primary, secondary or tertiary care.

**Results:**

We included 41 088 pregnant women. The groups differed significantly on maternal healthcare utilization. The late- or non-stoppers initiated prenatal care later and had less face-to-face consultations with primary care midwives during pregnancy. Compared to the non-smokers, the early- and late- or non-stoppers were statistically signficiantly more likely to be referred to the obstetrician during pregnancy and birth. Postpartum, the early- and late- or non-stoppers were statistically signficantly less likely to be referred to the obstetrician compared to the non-smokers.

**Conclusions:**

Although the early- and late- or non-stoppers initiated prenatal care later than the non-smokers, they did receive adequate prenatal care (according to the recommendations). The results suggest that not smoking during pregnancy may decrease the likelihood of referral to secondary or tertiary care. The large population of smokers being referred during pregnancy underlines the important role of the collaboration between healthcare professionals in primary and secondary or tertiary care. They need to be more aware of the importance of smoking as a medical and as a non-medical risk factor.

## Background

Research has shown that women who smoke during pregnancy have fewer contact moments with their midwives, and start prenatal care later compared to women who do not smoke during pregnancy, despite equal availability of prenatal care [1, 2]. Less contact moments with the midwife and later initiation of prenatal care for women who smoke during pregnancy has a twofold impact: both smoking during pregnancy and insufficient prenatal care are associated with an increased risk of low birth weight, neonatal death and stillbirth [3, 4]. Therefore, early initiation and adequate use of prenatal care should be pursued for women who smoke during pregnancy [5]. In the Netherlands it is common (more than 80%) that women start prenatal care with an independent primary care midwife [6]. If the midwife detects a serious risk factor during pregnancy or birth, she refers the woman to secondary or tertiary care [7]. A few studies reported an increase in referrals of pregnant women to secondary and tertiary care in the Netherlands, compared to years ago [8–11]. The main indications for this increase in referrals are fetal distress, a need for pain relief, meconium-stained amniotic fluid, and postpartum hemorrhage [8–11]. The influence of smoking status on the course of healthcare utilization has not been addressed in previous studies. Although we know that women who smoke during pregnancy begin later with prenatal care and pay less visits to the midwife [1, 2], we know little about their further maternal care trajectory. The association between smoking status, referrals and indications for referrals has not been studied before. Furthermore, the association between smoking cessation in the first trimester and referrals during the postpartum period are not examined in previous studies. The reported associations between smoking during pregnancy and increased risks of stillbirth, and operative birth interventions imply that women who smoke have more high-risk pregancies and require more specialized care [3, 12, 13]. Therefore, in the present study we aim to gain insight into the association between smoking status and healthcare utilization during pregnancy, birth, and six weeks postpartum. We aim to investigate initiation of maternal healthcare, number of contact moments with the midwife (contact in person or via telephone), frequency of referrals to primary, secondary and tertiary care, and the association of these factors with smoking status and the main indications for referrals. For this study we will use three groups: 1) pregnant women who do not smoke, 2) women who stopped smoking in the first trimester and 3) women who stopped smoking after the first trimester or who continued smoking during pregnancy. Insight into the course of healthcare utilization and referrals of pregnant women that differ in smoking status will increase our understanding of the impact of smoking on the maternal care trajectory. Based on the results of this study, recommendations for maternal care of women who smoke during pregnancy will be given.

## Methods

### Study design

We performed a cohort study based on data from the Midwifery Case Registration System (Verloskundig Casusregistratie Systeem, VeCaS) [14], initiated by the Midwifery Science Department from Zuyd University Maastricht, and the Midwifery Academy Amsterdam Groningen (AVAG). The VeCaS data consists of routinely extracted data from two different electronic healthcare registration systems: Orfeus and Vrumun [15, 16], both used by Dutch midwifery care practices. The database contains data from 44 primary midwifery care practices spread across the Netherlands. The data for this study were collected in the period from January 1, 2012 until December 31, 2019. All women in the database provided informed consent for the use of their anonymized data. The women in the VeCaS database are comparable to the national population of women in primary midwife-led care in the Netherlands [14]. We obtained ethical approval for use of the database from the regional Medical Research Ethics Committee Maastricht (nr 09–4–061) [14].

### Participants

For this study we selected women with singleton pregnancies, a known smoking status, a known parity and whose first appointment with their primary care midwife took place before 24 weeks of gestation. The threshold of 24 weeks was chosen because we wanted to have a sufficient amount of data of women’s pregnancy to be able to investigate their maternal healthcare utilization. Moreover, women who initiated care after 24 weeks of gestation likely started care at another midwifery care practice because they moved to another residence. We compared three groups of women based on smoking status, based on the categorisation of smoking behavior in the healthcare registration systems: non-smokers, early stoppers (women who stopped smoking during the first trimester), and late- or non-stoppers (women who stopped smoking after the first trimester or continued smoking during the entire pregnancy).

### Outcomes

From the electronic healthcare registration systems we extracted routinely collected data about the healthcare utilization of women during pregnancy, birth, and up to six weeks postpartum. We also extracted data regarding demographic characteristics: maternal age, Body Mass Index (BMI), ethnic background (Dutch, Western non Dutch, Non-Western or other), marital status (single, in a relationship), parity (nulliparous or multiparous), gestational age at birth (extremely preterm, very preterm, moderate to late preterm, term, and post term) [17], lifestyle characteristics (alcohol and drug consumption), socioeconomic status (SES), and smoking status. SES was calculated based on data from the Netherlands Institute for Social Research (SCP), including employment, education, and income level of the residential postal code area. Combined with the number of inhabitants per residential postal code area, based on data from Statistics Netherlands (CBS), we calculated national percentiles. We divided SES into low, middle, and high, based on percentile cut-off points: SES below the 25^th^ percentile we classified as low, and SES above the 75^th^ percentile as high. Smoking status was self-reported and divided into three categories: no smoking, stopped smoking in the first trimester, and stopped smoking after the first trimester or continued smoking during the entire pregnancy. Next, we collected data of pregnancy and birth characteristics: gestational diabetes mellitus, hypertensive disorder, placenta previa and fetal growth restriction [18–20]. Hypertensive disorder was defined based on the Dutch guideline as two blood pressure measurements after 20 weeks gestation with a diastolic pressure ≥ 90 and/or a systolic pressure ≥ 140 mmHg [21]. We also collected data regarding the mode of birth (spontaneous vaginal, instrumental, cesarean delivery), the maternal outcome postpartum hemorrhage > 1000 ml, and infant characteristics (sex, birth weight, and mortality). For mortality, we defined neonatal death as death during the first 28 days postpartum. We assessed maternal healthcare utilization based on the number of appointments with the primary care midwife, including specific details like week of gestation and mode of contact (face-to-face or via the telephone). Besides face-to-face contact, we decided to report telephone appointments since this is an important part of the provision of midwifery care. Dutch women can call the midwife for minor and for major issues. We defined initiation of care as the first personal appointment with the primary care midwife, indicated by both a registered blood pressure measurement and a known term date, expressed in gestational weeks.

Furthermore, we examined maternal healthcare utilization based on frequency of referrals from the primary care midwife to the general practitioner in primary care, or to the obstetrician, pediatrician or other specialist in secondary or tertiary care. We classified referrals to the obstetrician as either incidental referrals or referrals resulting in handover of care. The data in the healthcare registration system did not provide the ability to distinguish between referrals to secondary or tertiary care. From the data we derived the indications for referrals for each group, based on smoking status during pregnancy, birth, and postpartum.

### Statistical analysis

To report baseline characteristics we used descriptive statistics. To assess differences in baseline characteristics between the three groups of women based on smoking status we used chi-square tests. The data of initiation of care in gestational weeks, number of face-to-face contact moments, and number of telephonic contact moments were not normally distributed, we reported the medians (interquartile range) and calculated statistical differences between the three groups by means of Kruskal–Wallis tests. We performed multivariable logistic regression analyses to examine associations between smoking status and referrals during pregnancy, birth, and up to six weeks postpartum. Smoking status (non-smokers, early stoppers and late- or non-stoppers) was taken as independent variable, with non-smokers as reference category. Referrals to primary care (general practitioner) and secondary or tertiary care (incidental referrals, referrals resulting in handover of care by the obstetrician, referrals to the pediatrician, and referrals to an other specialist), were taken as dependent variables. We first calculated crude Odds Ratios (OR) with corresponding 95% Confidence Intervals (95% CI). We subsequently calculated adjusted Odds Ratios, and adjusted for possible confounders: BMI (continuous), maternal age (continuous), SES (using dummies with middle SES as reference category), and dichotomous variables: ethnicity (Dutch or non-Dutch) and parity (nulliparous or multiparous) [22, 23]. We did not control for pregnancy complications, because these are reason for referral and are causally associated with smoking behavior. Furthermore, we were not able to control for e-cigarette use during pregnancy because no women in our dataset reported using the e-cigarrette. Main indications for referrals are described according to a selection of the five main reasons for referral, based on smoking behavior during pregnancy, during birth, and up to six weeks postpartum. All data were analysed in SPSS version 26.0 (SPSS Inc., Chicago, IL, USA). A *p*-value of 0.05 was considered statistically significant.

## Results

**Fig. 1:**
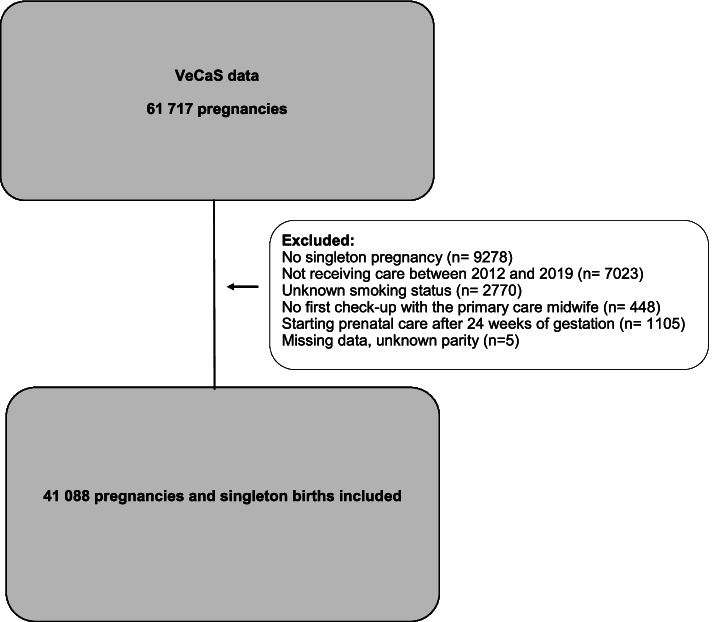
Flowchart of participants in the VeCaS data

From VeCaS we attained data of 61 717 women. From the dataset we excluded a total of 20 629 (33.4%) women. Reasons for exclusion were: no singleton pregnancy (*n* = 9278), not receiving care between 2012 and 2019 (i.e. these women received care after the inclusion period) (*n* = 7023), unknown smoking status (*n* = 2770), no first checkup with the primary care midwife (*n* = 448), starting prenatal care after 24 weeks of gestation (*n* = 1055) or unknown parity (*n* = 5) (illustrated in Fig. 1—Flowchart).

### Participants

In total, we included 41 088 pregnant women with a known smoking status and singleton pregnancies, and whose first appointment with the primary care midwife, before 24 weeks of gestation, took place between 2012 and 2019. Of this population, 83.0% were non-smokers, 7.8% were early stoppers, and 9.2% were late- or non-stoppers (Table 1). The three groups showed statistically significant differences on all maternal, birth and infant characteristics, except for the pregnancy complications gestational diabetes mellitus and placenta previa, and the infant characteristic mortality. Compared with the non-smokers, the late- or non-stoppers were of younger age, had a lower SES, were more often single, and showed a higher alcohol and drugs consumption. The late- or non-stoppers also showed higher proportions on maternal characteristics (preterm birth), pregnancy complications (fetal growth restriction) and infant characteristics (lower birthweight). The early stoppers were more often nulliparous, more likely to have hypertensive disorders, or had more often given birth by cesarean delivery.

**Table 1:** Maternal and infant characteristics by maternal smoking behavior during pregnancy; non-smokers, early stoppers and late- or non-stoppers

	**Total population** ***N* = 41 088**	**Non-smokers** ***n* = 34 102 (83.0%)**	**Early stoppers** ***n* = 3217 (7.8%)**	**Late- or non- stoppers** ***n* = 3769 (9.2%)**	**Statistical differences between subgroups that differed in smoking status**
	**N (%)**^**a**^	**n (%)**^**a**^	**n (%) **^**a**^	**n (%)**^**a**^	***p*-value**
**Maternal characteristics**					
**Maternal age (in years)**					≤ 0.001
< 20	249 (0.6)	118 (0.3)	54 (1.7)	77 (2.0)	
20–24	3209 (7.8)	1997 (5.9)	438 (13.6)	774 (20.5)	
25–29	12 499 (30.4)	9939 (29.1)	1200 (37.3)	1360 (36.1)	
30–34	16 660 (40.5)	14 577 (42.7)	1055 (32.8)	1028 (27.3)	
35–39	7331 (17.8)	6482 (19.0)	410 (12.7)	439 (11.6)	
> 39	1140 (2.8)	989 (2.9)	60 (1.9)	91 (2.4)	
**BMI**^**a**^					≤ 0.001
Underweight	1266 (3.1)	944 (2.8)	97 (3.0)	225 (6.0)	
Normal weight	24 251 (59.0)	20 521 (60.2)	1831 (56.9)	1899 (50.4)	
Pre-obesity	9542 (23.2)	7867 (23.1)	753 (23.4)	922 (24.5)	
Obesity class I	3496 (8.5)	2741 (8.0)	333 (10.4)	422 (11.2)	
Obesity class II	1170 (2.8)	915 (2.7)	103 (3.2)	152 (4.0)	
Obesity class II	270 (0.7)	210 (0.6)	27 (0.8)	33 (0.9)	
Missing	1093 (2.7)	904 (2.7)	73 (2.3)	116 (3.1)	
**Ethnic background**^**b**^					≤ 0.001
Dutch	31 271 (76.1)	25 965 (76.1)	2462 (76.5)	2844 (75.5)	
Western (non-Dutch)	2668 (6.5)	2107 (6.2)	275 (8.5)	286 (7.6)	
Non-Western	3892 (9.5)	3407 (10.0)	195 (6.1)	290 (7.7)	
Other	243 (0.6)	183 (0.5)	33 (1.0)	27 (0.7)	
Missing	3014 (7.3)	2440 (7.2)	252 (7.8)	322 (8.5)	
**Socio economic status**^**c**^					
Low	12 942 (31.5)	9874 (29.0)	1170 (36.4)	1898 (50.4)	≤ 0.001
Middle	20 841 (50.7)	17 643 (51.7)	1626 (50.5)	1572 (41.7)	
High	6992 (17.0)	6325 (18.5)	394 (12.2)	273 (7.2)	
Missing	313 (0.8)	260 (0.8)	27 (0.8)	26 (0.7)	
**Marital status**					≤ 0.001
Single	926 (2.3)	515 (1.5)	117 (3.6)	294 (7.8)	
In a relationship	39 040 (95.0)	32 832 (96.3)	2964 (92.1)	3244 (86.1)	
Missing	1122 (2.7)	755 (2.2)	136 (4.2)	231 (6.1)	
**Parity**					≤ 0.001
Nulliparous	19 300 (47.0)	15 616 (45.8)	1989 (61.8)	1695 (45.0)	
Multiparous	21 788 (53.0)	18 486 (54.2)	1228 (38.2)	2074 (55.0)	
**Gestational age at birth**					≤ 0.001
Extremely preterm (< 28 weeks)	337 (0.8)	272 (0.8)	25 (0.8)	40 (1.1)	
Very preterm (28–32 weeks)	196 (0.5)	159 (0.5)	14 (0.4)	23 (0.6)	
Moderate to late preterm (32–37 weeks)	1535 (3.7)	1184 (3.5)	138 (4.3)	213 (5.7)	
Term (37–42)	36 109 (87.9)	30 136 (88.4)	2783 (86.5)	3190 (84.6)	
Postterm (≥ 42 weeks)	547 (1.3)	478 (1.4)	41 (1.3)	28 (0.7)	
Missing	2364 (5.8)	1873 (5.5)	216 (6.7)	275 (7.3)	
**Lifestyle**					
**Alcohol consumption**^**d**^					≤ 0.001
No	24 367 (59.3)	20 310 (59.6)	1875 (58.3)	2182 (57.9)	
Yes	241 (0.6)	183 (0.5)	28 (0.9)	30 (0.8)	
Stopped after a positive pregnancy test	611 (1.5)	448 (1.3)	106 (3.3)	57 (1.5)	
Missing	15 869 (38.6)	13 161 (38.6)	1208 (37.6)	1500 (39.8)	
**Drug consumption**^**e**^					≤ 0.001
No	22 700 (55.2)	18 949 (55.6)	1767 (54.9)	1984 (52.6)	
Yes	182 (0.4)	56 (0.2)	43 (1.3)	83 (2.2)	
Stopped recently	150 (0.4)	25 (0.1)	56 (1.7)	69 (1.8)	
Missing	18 056 (43.9)	15 072 (44.2)	1351 (42.0)	1633 (43.3)	
**Pregnancy complications**^f^					
Gestational Diabetes Mellitus	1385 (3.4)	1142 (3.3)	106 (3.3)	137 (3.6)	0.63
Hypertensive disorder	2311 (5.6)	1925 (5.6)	220 (6.8)	166 (4.4)	≤ 0.001
Placenta previa	212 (0.5)	177 (0.5)	17 (0.5)	18 (0.5)	0.94
Fetal growth restriction	1489 (3.6)	1055 (3.1)	131 (4.1)	303 (8.0)	≤ 0.001
**Mode of birth characteristics**					≤ 0.001
Spontaneous vaginal birth	30 327 (73.8)	25 358 (74.4)	2223 (69.1)	2746 (72.9)	
Instrumental birth	3212 (7.8)	2639 (7.7)	301 (9.4)	272 (7.2)	
Cesarean delivery	4060 (9.9)	3317 (9.7)	379 (11.8)	364 (9.7)	
Other	695 (1.7)	578 (1.7)	61 (1.9)	56 (1.5)	
Missing	2794 (6.8)	2210 (6.5)	253 (7.9)	331 (8.8)	
**Maternal Outcomes**					≤ 0.001
Postpartum hemorrhage > 1000 ml	1227 (3.0)	1032 (3.0)	104 (3.2)	91 (2.4)	
**Infant characteristics**					
**Sex**					0.005
Female	18 819 (45.8)	15 688 (46.0)	1383 (43.0)	1748 (46.4)	
Male	19 837 (48.3)	16 490 (48.4)	1612 (50.1)	1735 (46.0)	
Missing	2432 (5.9)	1924 (5.6)	222 (6.9)	286 (7.6)	
**Birthweight (grams)**					≤ 0.001
≤ 1500	415 (1.0)	323 (0.9)	33 (1.0)	59 (1.6)	
1501–2500	1271 (3.1)	854 (2.5)	120 (3.7)	297 (7.9)	
2501–3500	18 052 (43.9)	14 519 (42.6)	1438 (44.7)	2095 (55.6)	
3501–4500	18 055 (43.9)	15 709 (46.1)	1342 (41.7)	1004 (26.6)	
≥ 4501	750 (1.8)	671 (2.0)	55 (1.7)	24 (0.6)	
Missing	2545 (6.2)	2026 (5.9)	229 (7.1)	290 (7.7)	
**Mortality**					0.125
Fetal death	273 (0.7)	218 (0.6)	18 (0.6)	37 (1.0)	
Neonatal death	43 (0.1)	37 (0.1)	2 (0.1)	4 (0.1)	

Percentages may not add up to 100% due to rounding

^a^BMI classified as: Underweight (< 18.5), Normal Weight (18.5–24.9), Pre-obesity (25.0–29.9), Obesity class I (30.0–34.9), Obesity class II (35.0–39.9), Obesity class III (> 40)

^b^Ethnic background classified as: Dutch, Western non-Dutch (European), Non-Western (African, Asian, Turkish, Moroccan) and other migration backgrounds

^c^Socioeconomic status classified as: Low (below the 25^th^ percentile), Middle (between the 25^th^ and 50^th^ percentile), High (above the 75^th^ percentile)

^d^Alcohol consumption: No (no alcohol use), Yes (ranging from occassional use to daily use), Stopped after a positive pregnancy test

^e^ Drugs consumption: No (no drug use), Yes (ranging from occasional use to daily use), Stopped recently

^f^Multiple pregnancy complications are possible, therefore they do not add up to 100%

### Initiation of maternal healthcare

The three groups differed significantly regarding the gestational age at initiation of maternal healthcare, number of face-to-face visits, and the number of contact moments by telephone with the midwife (p ≤ 0.001) (Table 2). The late- or non-stoppers started maternal care significantly later, at a median of 9.4 weeks of pregnancy (IQR 8.0–11.2), than the early stoppers, at a median of 9.1 weeks (IQR 8.0–10.7). Although the three groups differed significantly on the number of face-to-face visits, the medians and interquartile ranges were mostly similar. Based on the lower quartile, the late- or non-stoppers (IQR 8–14) had fewer face-to-face visits with the primary care midwife than the non-smokers (IQR 9–14) and early stoppers (IQR 9–14). With regard to the number of telephonic consultations, the non-smokers seem to have less consultations via telephone (IQR 0–3) than the early- (IQR 0–4) and the late- or non-stoppers (IQR 0–4) based on the upper quartile.

**Table 2:** Initation and frequency of antenatal consultations with the midwife by smoking behavior during pregnancy

	**Total** ***N* = **41 088	**Non-smokers** ***n* = **34 102	**Early stoppers** ***n* = **3217	**Late- or non-stoppers** ***n* = **3769	***p*-value**
	**Median (Interquartile Range)**	**Median (IQR)**	**Median (IQR)**	**Median (IQR)**	
Initatition of maternal healthcare (in weeks of gestation)	9.4 (8.1–11.0)	9.4 (8.1–11.0)	9.1 (8.0–10.7)	9.4 (8.0–11.2)	≤ 0.001
Number of antenatal face-to-face visits (regular consultations in person)	12.0 (9.0–14.0)	12.0 (9.0–14.0)	12.0 (9.0–14.0)	12.0 (8.0–14.0)	≤ 0.001
Number of contact moments via telephone	2.0 (0.0–4.0)	2.0 (0.0–3.0)	2.0 (0.0–4.0)	2.0 (0.0–4.0)	≤ 0.001

### Referrals

The results of referrals are indicated per stadium of pregnancy, birth, and six weeks postpartum. Concerning referrals to the obstetrician during pregnancy, the majority were referred for an incidental consult (40.0%), followed by handover of care (33.1%). Of the late- or non-stoppers, 40.5% were referred to the obstetrician, resulting in handover of care during pregnancy. Across all three groups, women were least often referred to an other specialist (0.6%) (Table 3). Compared to the non-smokers, both the early stoppers and the late- or non-stoppers were statistically significantly more likely to be referred to the obstetrician for an incidental referral, with Crude ORs of 1.15 (95%CI 1.07–1.24) and 1.24 (95%CI 1.16–1.33) respectively. This association remained significant after adjustment for confounders: aOR 1.11 (95% 1.03–1.20) for the early stoppers and 1.20 (95%CI 1.11–1.29) for the late- or non-stoppers. Compared to non-smokers, the early stoppers and late- or non-stoppers also were statistically significantly more likely to be referred to the obstetrician resulting in handover of care, with Crude ORs of 1.58 (95%CI 1.45–1.73) and 1.64 (95% CI 1.52–1.78), respectively. After adjusting for confounders, this remained significant: aOR 1.49 (95%CI 1.36–1.63) for early stoppers and aOR 1.58 (95%CI 1.45–1.73) for the late- or non-stoppers. During birth, for most women who had been referred to the obstetrician (22.6%), the referral resulted in handover of care. Compared to the non-smokers, the late- or non-stoppers were statistically significantly more likely to be referred to the obstetrician resulting in handover of care: OR 1.56 (95%CI 1.42–1.72) and OR 1.41 (95%CI 1.29–1.54), respectively. After adjusting for confounders, the associations remained statistically significant: aOR 1.30 (95%CI 1.17–1.44) for early stoppers and aOR 1.40 (95%CI 1.27–1.55) for late- or non-stoppers. Referrals to the general practitioner, pediatrician and other specialists were not taken into account, since these referrals were not applicable during birth.

Up to six weeks postpartum, most women (1.7%) were referred to the obstetrician resulting in handover of care; followed by referrals to the pediatrician (1.5%). A small number of women were referred to the general practitioner (*n* = 33) or an other specialist (*n* = 21). The late- or non-stoppers were statistically significantly less likely to be referred for an incidental referral to the obstetrician: OR 0.69 (95%CI 0.49–0.99), compared to the non-smokers. This association remained statistically significant after adjusting for confounders: aOR 0.69 (0.47–0.99). The early stoppers were statistically signficiantly less likely to be referred to the obstetrician resulting in handover of care: OR 0.69 (95%CI 0.47–0.99), compared to the non-smokers. After adjusting for confounders, this association remained statistically significant, aOR 0.63 (95%CI 0.43–0.93). Due to the low frequencies of referrals, we omitted the general practitioner and the other specialist from the calculation.

**Table 3:** Association between smoking status and referrals to maternal care professionals

	**Proportion N (%)**	**Crude OR (95% CI)**	**Adjusted OR (95% CI)**^a^
**Referral outcomes**			
**During pregnancy**			
**General practitioner**	432 (1.1)		
Non-smokers	363 (1.1)	Ref	Ref
Early stoppers	30 (0.9)	0.88 (0.60–1.27)	0.88 (0.59–1.32)
Late- or non-stoppers	39 (1.0)	0.97 (0.70–1.36)	0.92 (0.64–1.32)
**Obstetrician (incidental)**	16 436 (40.0)		
Non-smokers	13 378 (39.2)	Ref	Ref
Early stoppers	1374 (42.7)	**1.15 (1.07–1.24)**^**b**^	**1.11 (1.03–1.20)**
Late- or non-stoppers	1684 (44.7)	**1.24 (1.16–1.33)**	**1.20 (1.11–1.29)**
**Obstetrician (handover of care)**	13 612 (33.1)		
Non-smokers	10 844 (31.8)	Ref	Ref
Early stoppers	1242 (38.6)	**1.58 (1.45–1.73)**	**1.49 (1.36–1.63)**
Late- or non-stoppers	1526 (40.5)	**1.64 (1.52–1.78)**	**1.58 (1.45–1.73)**
**Other specialist**	237 (0.6)		
Non-smokers	194 (0.6)	Ref	Ref
Early stoppers	25 (0.8)	1.37 (0.90–2.08)	1.50 (0.98–2.30)
Late- or non-stoppers	18 (0.5)	0.84 (0.52–1.36)	1.03 (0.63–1.68)
**During birth**			
**Obstetrician (incidental)**	340 (0.8)		
Non-smokers	286 (0.8)	Ref	Ref
Early stoppers	22 (0.7)	0.86 (0.55–1.33)	0.79 (0.50–1.25)
Late- or non-stoppers	32 (0.8)	1.10 (0.76–1.59)	0.94 (0.62–1.43)
**Obstetrician (handover of care)**	9298 (22.6)		
Non-smokers	7540 (22.1)	Ref	Ref
Early stoppers	851 (26.5)	**1.56 (1.42–1.72)**	**1.30 (1.17–1.44)**
Late- or non-stoppers	907 (24.1)	**1.41 (1.29–1.54)**	**1.40 (1.27–1.55)**
** < 6 weeks postpartum**			
**General practitioner**	33 (0.1)		
Non-smokers	27 (0.1)	Ref	Ref
Early stoppers	4 (0.1)	NA	NA
Late- or non-stoppers	2 (0.1)	NA	NA
**Obstetrician (incidental)**	541 (1.3)		
Non-smokers	468 (1.4)	Ref	Ref
Early stoppers	40 (1.2)	0.96 (0.69–1.32)	0.88 (0.62–1.24)
Late- or non-stoppers	33 (0.9)	**0.69 (0.49–0.99)**	**0.69 (0.47–0.99)**
**Obstetrician (handover of care)**	682 (1.7)		
Non-smokers	606 (1.8)	Ref	Ref
Early stoppers	30 (0.9)	**0.69 (0.47–0.99)**	**0.63 (0.43–0.93)**
Late- or non-stoppers	46 (1.2)	0.89 (0.65–1.20)	0.82 (0.59–1.13)
**Pediatrician**	618 (1.5)		
Non-smokers	525 (1.5)	Ref	Ref
Early stoppers	39 (1.2)	0.79 (0.57–1.09)	0.73 (0.51–1.04)
Late- or non-stoppers	54 (1.4)	0.93 (0.70–1.23)	0.92 (0.68–1.24)
**Other specialist**	21 (0.1)		
Non-smokers	20 (0.1)	NA	NA
Early stoppers	1 (0.0)	NA	NA
Late- or non-stoppers	-	NA	NA

^a^Multivariable logistic regression models were adjusted for maternal age, BMI, socioeconomic status, parity, and ethnicity

^b^Associations in bold are statistically significant p ≤ 0.05

### Indications for referral

**Fig. 2:**
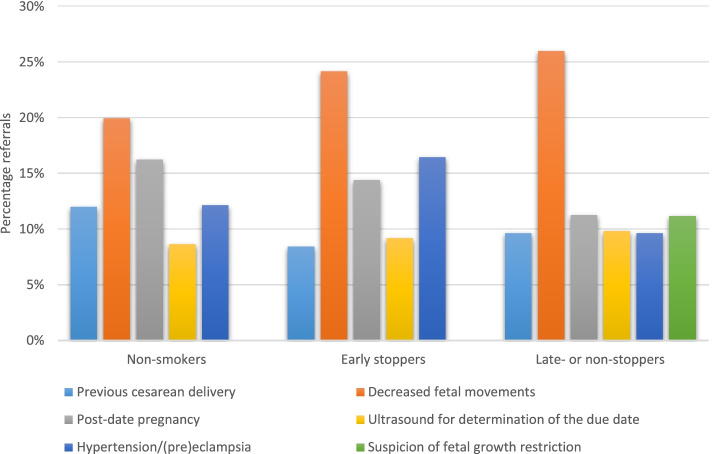
The five main indications for referral during pregnancy

**Fig. 3:**
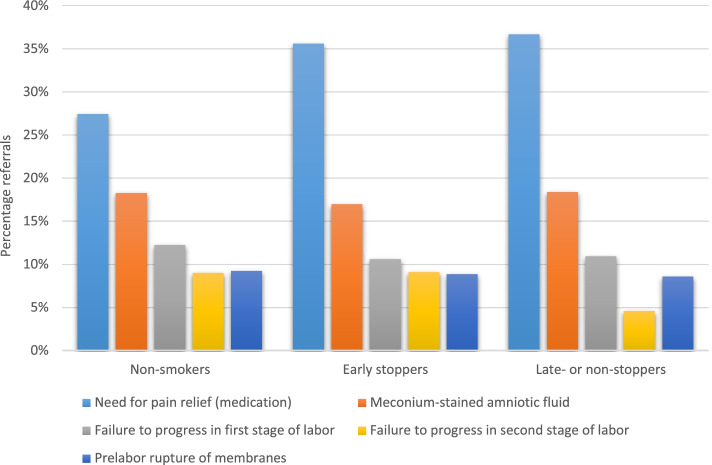
The five main indications for referral during birth

**Fig. 4:**
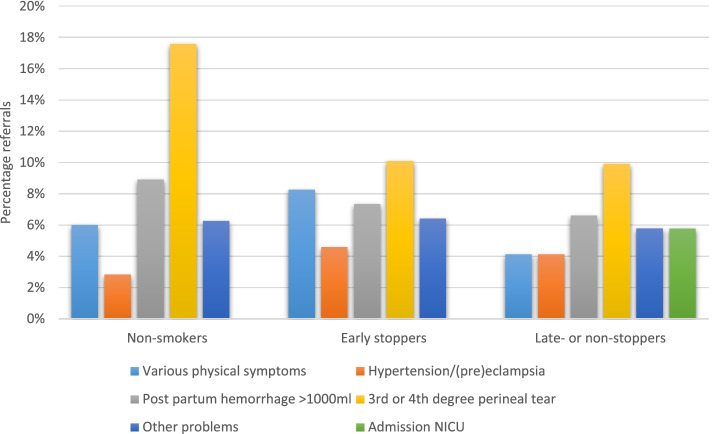
The five main indications for referral postpartum

During pregnancy, the five main indications for referral were decreased fetal movements, post-date pregnancy, hypertension/(pre)eclampsia, previous cesarean delivery and ultrasound for determination of the due date (Fig. 2). The main indications for the non-smokers and the early stoppers were similar; only the ranking differed. The main indications for referral were the same for the late- or non-stoppers, except for the indication suspicion of fetal growth restriction. Instead of five indications we reported six indications for referral for the late- or non-stoppers, because the percentages of some indications (previous cesarean delivery and hypertension/(pre)eclampsia) were equal. During birth, the main indications for referral were: need for pain relief, meconium-stained amniotic fluid, failure to progress in first stage of labor, failure to progress in second stage of labor, and prelabor rupture of membranes (Fig. 3). The late- or non-stoppers were less often referred for failure to progress in second stage of labor compared to the other groups. Postpartum, the main indications for referral were 3^rd^ or 4^th^ degree perineal tear, post partum hemorrhage > 1000 ml, various physical symptoms, and other problems (Fig. 4). These indications were the same for the non-smokers and the early stopppers, the late- or non-stoppers also had admission to the neonatal intensive care unit (NICU) as main indication for referral.

## Discussion

### Main findings

The aim of this study was to investigate maternal healthcare utilization for three groups of women, classified according to smoking status: non-smokers, early stoppers, and late- or non-stoppers. These groups showed statistically significant differences in the gestational age at initiation of maternal healthcare and number of contact moments, and in referrals during pregnancy, birth, and postpartum. First, we found that the late- or non-stoppers initiate maternal care later, and have fewer face-to-face visits with the midwife, compared to non-smokers or early stoppers. The non-smokers have less telephonic consultations with the midwife compared to the early stoppers and the late- or non-stoppers. Second, compared to the non-smokers, the early stoppers and the late- or non-stoppers were statistically significantly more likely to be referred to the obstetrician during pregnancy and birth. This seems to be reversed postpartum, the early stoppers and the late- or non-stoppers were statistically significantly less likely to be referred to the obstetrician compared to the non-smokers. Third, we identified the main indications for referral during pregnancy, birth, and postpartum; these were mostly similar for the three groups.

### Strenghts and limitations

This study has some strenths and limitiations to consider when interpreting the results. One strength is its use of primary midwifery care registration data; the women in the VeCaS data are comparable to the Dutch national population of women in primary midwife-led care [14]. The observed prevalence of smokers is a bit higher than that of the average population in the Netherlands [24], but the observed rates of referral are comparable to those in a previous Dutch study [9]. In addition, the large sample size made it possible to investigate differences in maternal healthcare utilization among the three groups of smokers. A first limitation of our study is that referrals to the general practitioner and the other specialist may be underreported, as pregnant women may visit general practitioners and an other specialist without referral by the midwife. The results of a previous study, using general practitioner registration data, imply that 65% of pregnant women contact their general practitioner [25]. This percentage is much higher than the 1.1% of women in our study who were referred to the general practitioner. The previous study offers a possible explanation for this difference by stating that pregnant women might contact their general practitioner first before they contact their midwife, because women are more familiar with their general practitioner or do not know whether a symptom is pregnancy related. It could also be that referrals to the general practitioner are underreported in VeCaS, since pregnant women can contact the general practitioner by themselves and do not need interference from a midwife. Second, in our study we have not taken the number of cigarettes into account. The number of cigarettes smoked is known to influence health outcomes of the baby [26], and thereby possibly maternal healthcare utilization. Third, we were not able to make a distinction between women who stopped smoking after the first trimester and women who continued smoking, because the VeCaS data only contains data about the three groups of smokers. Fourth, we do not have data of women who find out they were pregnant during birth, although this will be a very small percentage of women. Data of these women is available in Perined, a Dutch data registry that captures primary, secondary and tertiary care data. We decided not to use Perined data for our study because smoking status is heavily underreported in this dataset [27]. Lastly, we are aware that the association between smoking behavior and healthcare utilization is complex (e.g. smoking behavior is also related to other lifestyle factors like diet and physical activity). In our study we decided to take a pragmatic approach, focusing only on the association between smoking behavior and healthcare utilization.

### Interpretation

Our finding that late- or non-stoppers initiate prenatal care later and have fewer face-to-face visits with the midwife could possibly be explained by the pregnancy recognition time. Previous studies reported that smoking during pregnancy is associated with unplanned pregnancy [28], leading to a later initiation of care and less prenatal visits [29]. Although the late- or non-stoppers initiate maternal care significantly later, our results do not indicate that either early stoppers or late- or non- stoppers have inadequate use of care. This finding partly corresponds to the results of previous studies, reporting that women who smoke during pregnancy initiate prenatal care later [1, 2, 30, 31]. In accordance with the recommendations in the Dutch guideline, the early stoppers and late- or non-stoppers in our study have a median initiation of care before 10 weeks of gestation, and a median of 12 face-to-face visits with the midwife [32]. Therefore, the late- or non-stoppers still meet the recommendations, despite the statistically significant differences between the groups on their use of maternal care; the differences are therefore not clinically relevant. This also applies to the number of face-to-face visits and telephonic consultations. The statistically significant differences, despite the similarities in medians, could be explained by skewed distributions and the large group sizes. We found that compared to the non-smokers, women who are early stoppers or late- or non-stoppers have more telephone consultations with the primary care midwife. Telephone consultations are often peformed at the initiative of the pregnant women themselves, and are used by midwives to provide education, support, and triage [33]. Based on the telephone consults, midwives can decide to refer women to secondary or tertiary care [33]. This higher number of telephonic consultations may be related to the increased referral rates of early- and late- or non-stoppers. Secondly, we found that pregnant women who smoke at the beginning of pregnancy, both early stoppers- and late- or non-stoppers, are more likely to be referred to the obstetrician during pregnancy and birth. Previous studies reported a positive effect of early smoking cessation on birth outcomes [26, 34], implying that early stoppers would be less likely to be referred to the obstetrician than late- or non-stoppers. However, we did not find large differences in referrals between the early stoppers and the late- or non-stoppers. This finding could be explained by the relatively higher proportions of fetal growth restriction, preterm births, and lower birth weights of infants that we found in our population of early stoppers and late- or non-stoppers, compared to the non-smokers. These are all possible negative health consequences of smoking during pregnancy [3], and indications for referral to secondary or tertiary care [35]. Postpartum, the association between smoking behavior during pregnancy and birth, and referrals, seems to be reversed. The early- and late- or non-stoppers are less likely to be referred to the obstetrician. A possible explanation may be the higher rates of referral to the obstetrician during pregnancy and birth, resulting in the handover of care. This might imply that women with higher risk pregnancies are already in secondary or tertiary care and therefore are not referred again postpartum. Therefore, additional referral postpartum is not applicable. This could also be illustrated by the differences in mode of birth between the groups [8, 9]. The early stoppers more often have an instrumental birth or cesarean delivery, implying that they already receive care from an obstetrician during birth in secondary or tertiary care. Third, among the three groups we did not find differences in indications large enough to explain the significant differences in referral rates. Women’s smoking behavior might be unrelated to the main indications for referral. Our identified indications for referrals are generally comparable to those reported in previous studies [8, 9]. Nevertheless, despite these similar indications for referral in the three groups, our results show that women who smoke during pregnancy are more often referred to the obstetrician. Considering the negative health effects of smoking [3], it may be that, for smokers, the indications for referral involve more serious health consequences, leading to higher referral rates. Furthermore, other concomitant non-medical risk factors associated with smoking during pregnancy may possibly influence the higher referral rates, such as a lower SES, lower education level, higher alcohol and drug use, and higher levels of anxiety and depression [5, 36]. These factors are also associated with adverse pregnancy outcomes, such as small for gestational age, preterm delivery and stillbirth [37–39], which could lead to higher referral rates to secondary and tertiary care. The higher prevalence of these factors in women who smoke during pregnancy might imply that they form a vulnerable group with a higher risk for adverse pregnancy outcomes, requiring more specialized care. In the Netherlands, special care paths are available that describe how vulnerable pregnant women can receive specialized care during pregnancy. The care for vulnerable pregnant women also involves consultations in which interdisciplinary healthcare professionals collaborate in the care for vulnerable women. However, a previous study reports that interdisciplinary care for vulnerable pregnant women still should be improved [40]. The study recommends that interdisciplinary healthcare professionals should take both medical risk factors (pregnancy outcomes) and non-medical risk factors (such as a low SES and poor lifestyle behaviors) into account [40]. The results of our study indicate the importance of smoking as a risk factor, related to both medical and non-medical factors, that should be taken into account in the care for (vulnerable) pregnant women.

## Conclusion

In this study we aimed to gain insight into similarities and differences in maternal healthcare utilization for women who do not smoke during pregnancy, early stoppers, and late- or non-stoppers. Our results indicate that the three groups differ in their utilization of care. However, although the late- or non-stoppers initiate maternal care later, their use of prenatal care is adequate. Furthermore, women who smoke at the beginning of pregnancy are more often referred to the obstetrician during pregnancy and birth than non-smokers. The large population of smokers being referred during pregnancy underlines the important role of the collaboration between healthcare professionals in primary and secondary or tertiary care. They need to be more aware of the importance of smoking as a medical and as a non-medical risk factor. More research is needed on the influence of the amount of cigarettes smoked on maternal healthcare use. We recommend future studies to make a distinction between women who stopped smoking after the first trimester and women who continued smoking to investigate the association on referrals to secondary or tertiary care.

## Data Availability

The data that support the findings of this study are available from Zuyd University Maastricht or the Midwifery Academy Amsterdam Groningen but restrictions apply to the availability of these data, which were used under license for the current study, and so are not publicly available. Data are however available from the authors upon reasonable request and with permission of Zuyd University Maastricht and the Midwifery Academy Amsterdam Groningen.
